# Systemic Inflammatory Response and Severe Thrombocytopenia after Endovascular Thoracic Aortic Aneurysm Repair

**DOI:** 10.1155/2017/3018919

**Published:** 2017-01-05

**Authors:** Valentina Silvestrin, Stefano Bonvini, Michele Antonello, Franco Grego, Roberto Vettor, Marco Rossato

**Affiliations:** ^1^Department of Medicine-DIMED, Clinica Medica 3, University Hospital of Padova, Padova, Italy; ^2^Department of Cardiac, Thoracic and Vascular Sciences, Division of Vascular and Endovascular Surgery, University of Padova, Padova, Italy

## Abstract

After Endovascular repair of thoracic aortic aneurysm, a systemic inflammatory response, named postimplantation syndrome, can develop. This syndrome is characterized by fever, leukocytosis, and elevated CRP plasma levels and its pathogenetic mechanisms are still unknown. Although this syndrome generally resolves within few days, some patients develop a persisting severe inflammatory reaction leading to mild or severe complications. Here we describe the case of a male patient who developed postimplantation inflammatory syndrome and severe thrombocytopenia after endovascular repair of thoracic aortic aneurysm. Treatment with prednisone (50 mg/bid) for two weeks did not improve the clinical and laboratory findings. We utilized danazol, a weak androgen that has been shown to be effective in the treatment of immune and idiopathic thrombocytopenic purpura, and after 12 days of treatment with danazol (200 mg/bid), the patient improved progressively and platelet number increased up to 53,000/*μ*L. Patients undergoing endovascular repair of thoracic aortic aneurysm should be carefully monitored for the development of postimplantation syndrome. This clinical condition is relatively common after the endovascular repair of aortic aneurysm but is rarely observed after endovascular repair of thoracic aortic aneurysms. The different known therapeutical approaches are still empiric, with reported beneficial effects with the use of NSAID, corticosteroids, and danazol.

## 1. Introduction

Aortic aneurysms are a relatively common disease that can lead to potentially fatal consequences [1, 2]. Endovascular repair of thoracic aortic aneurysms (TEVAR) is a minimally invasive surgery for their treatment, presenting many advantages over the open repair [3, 4]. After the correction of aortic aneurysm by TEVAR, a systemic inflammatory response, named postimplantation syndrome (PIS), can develop, being characterized by fever, leukocytosis, and elevated CRP plasma levels [5–8]. PIS is a relatively common complication after the endovascular repair of aortic aneurysm with an incidence of 3–60% for abdominal aortic aneurysm, although there are no conclusive data on the incidence of PIS for thoracic aortic aneurysms [5]. The clinical manifestations of PIS could be considered as a systemic inflammatory response syndrome (SIRS), since PIS fulfills at least two criteria of SIRS, in accordance with the International Sepsis Definitions Conference [8]. PIS after TEVAR is generally well tolerated, resolving within few days without any serious consequence. However, some patients develop a severe inflammatory reaction after TEVAR which could persist during the first month. This condition may lead to mild or severe SIRS state, possibly causing severe complications, such as pulmonary dysfunction, cardiovascular events, renal insufficiency, and multiple organ failure [3, 4, 6]. The lack of a well-recognized pathogenesis of PIS humpers a specific pharmacological treatment, although, as for SIRS, the use of nonsteroidal anti-inflammatory drugs in mild forms and of corticosteroids in severe forms has been suggested, together with intensive care assistance [3, 4, 6].

We describe the case of a male patient who developed postimplantation inflammatory syndrome and severe thrombocytopenia after endovascular repair of thoracic aortic aneurysm.

## 2. Case Presentation

**Figure 1:**
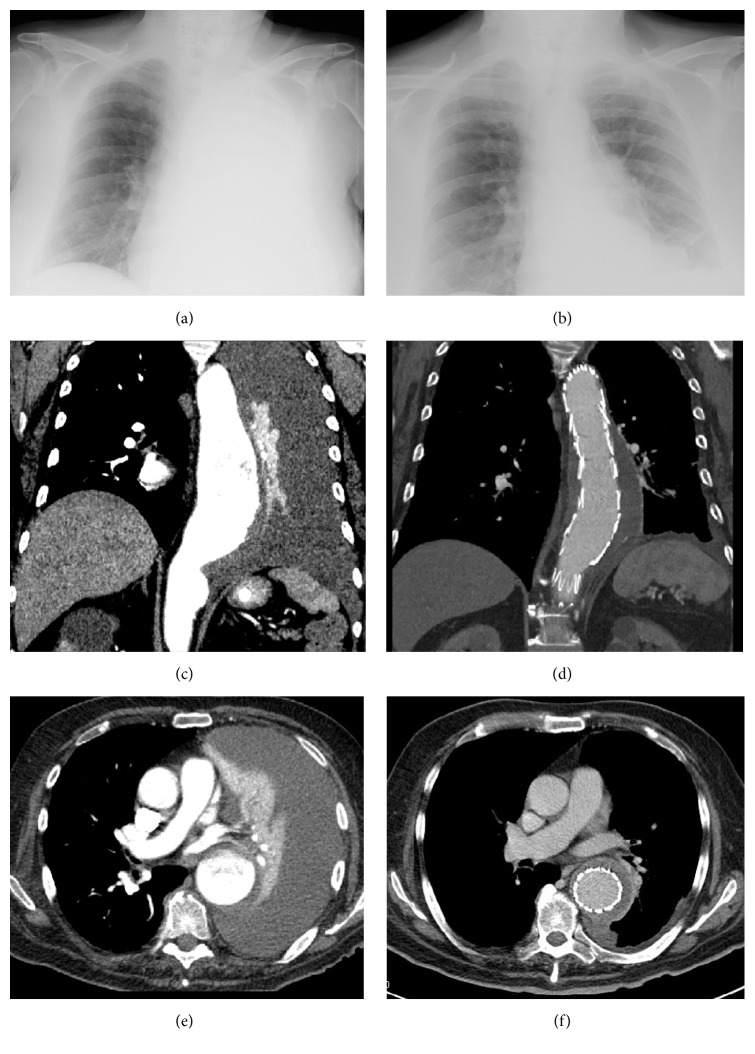
Chest X-ray before (a) and after (b) endovascular repair of thoracic aortic aneurysm (TEVAR) showing the complete opacity of the left lung field due to aneurysm rupture within the pleura (a) and the complete resolution after blood drainage (b). CT-scan before (c and e) and after (d and f) endovascular repair of thoracic aortic aneurysm by TEVAR.

A 92-year-old man was admitted to emergency department for dyspnea and asthenia. His medical history included chronic kidney disease, noncritical carotid artery stenosis, chronic obstructive pulmonary disease (GOLD stage I), and arterial hypertension. After admission, the patient reported epigastric pain radiating to the back many weeks previously. He showed normal body temperature (36.6°C) and heart rate (90 beats/min), with elevated arterial pressure (170/100 mmHg) and a pulse oximetry of 90%. On physical examination, he presented an area of dullness at the lower half of the left hemithorax with abolished vesicular murmur in the same area. Peripheral arterial pulses were symmetric. Routine laboratory data showed normocytic-normochromic anemia (Hb 105 g/L) with increased reticulocyte percentage (2.40%), neutrophilic and monocytic leukocytosis (WBCs 11,120/*μ*L, neutrophils 9,340/*μ*L, and monocytes 1,390/*μ*L), and an increase in inflammatory markers (CRP 251 mg/L). The chest X-ray was compatible with left pleural effusion. The patient was initially treated with oxygen therapy, intravenous corticosteroid, and antibiotic treatment with partial improvement of the dyspnea and increase in oxygen saturation. Eleven days after the admission, the patient reported worsening of the dyspnea and a second chest X-ray showed complete opacity of the left lung field (Figure 1).

Contrast chest CT-scan revealed a descending thoracic and proximal abdominal aortic aneurysm, with maximal axial diameter of 7 cm, presenting a thick parietal plaque (17 mm) with ulcerative aspects and hyperdensity on the left anterolateral side compatible with intramural hematoma (Figure 1). The patient was promptly operated on by vascular surgeons with an endovascular repair of the thoracic aortic aneurysm (TEVAR) by the placement of endoprosthesis (Zenith TX2 TAA endovascular graft, Cook Medical, Bloomington, IN, USA). Three weeks after surgical treatment, the patient developed progressive thrombocytopenia with lowest value of 14,000/*μ*L: these values were not correlated to low molecular weight heparin treatment, since the platelet factor 4/heparin antibodies were negative, excluding a heparin induced thrombocytopenia. A disseminated intravascular coagulation was also excluded (normal INR, fibrinogen, and antithrombin-III) [9]. Laboratory findings showed leukocytosis during the first postoperative day (WBCs 14,180/*μ*L) with WBCs up to 18,000/*μ*L during the second postoperative day; furthermore, there was an increase in CRP values. The patient presented fever with a peak of 38°C despite negative blood, urine, and bronchial aspirate cultures. Plasma concentrations of TNF-*α* and IL-6 were elevated, respectively (25.4 ng/L (normal values: 0.0–8.1 ng/L) and 37.9 ng/L (normal values: 0.0–5.9 ng/L)). As empiric therapeutic approach to thrombocytopenia, we first started corticosteroid therapy (prednisone 50 mg/bid) for two weeks without significant improvement in platelet count. After this unsuccessful therapy, we used danazol, a weak androgen that has been shown to be effective in the treatment of immune and idiopathic thrombocytopenic purpura and in thrombocytopenia in patients with myelodysplastic syndrome [10, 11]. After 12 days of treatment with danazol (200 mg/bid), PLT values increased up to 53,000/*μ*L. The patient was discharged and admitted to a long-term care facility.

## 3. Discussion

Endovascular repair of thoracic aortic aneurysm is a minimally invasive surgery presenting many advantages over the open repair [3, 4]. After the correction of aortic aneurysm by TEVAR, a systemic inflammatory response, named postimplantation syndrome (PIS), can develop, being characterized by fever, leukocytosis, and elevated CRP plasma levels [5–8]. The pathogenetic mechanisms of PIS are still unknown: fever and leukocytosis have been attributed to an activation of the inflammatory pathways induced by the aneurysm exclusion and mediated by the release of several inflammatory cytokines including TNF-*α*, IL-1 *α*, IL-1*β*, and IL-6 [5–7]. IL-6 is secreted by mononuclear phagocytes, endothelial cells, and lymphocytes T, and its secretion is due to ischemia/reperfusion injury resulting by the surgical procedure and exposition of leukocytes to the components of the prosthesis.

To this regard, the impact of PIS on patient outcome is still unknown but the systemic inflammatory response could be responsible for an increased cardiovascular morbidity, since the intensity of inflammation seems to be correlated with the presence of cardiovascular or any other adverse event during the first month after the procedure [4]. Furthermore, Arnaoutoglou et al. demonstrated that PIS was the only independent predictor of cardiovascular or other adverse events after the first 30 days and during the first year of follow-up after endovascular aneurysm repair for AAA [4, 12].

Then patients with PIS may require closer surveillance during the first year after TEVAR [12], although more studies are needed to establish such a relation.

A specific pharmacological treatment for PIS has not been suggested yet, although, as for SIRS, the use of nonsteroidal anti-inflammatory drugs in mild forms and of corticosteroids in severe forms has been suggested, together with intensive care assistance [3, 4, 6]. In the present case report, after TEVAR for TAA, our patient developed a clinical syndrome fulfilling the diagnostic criteria for PIS. To this regard, the large majority of cases of PIS have been described after EVAR for AAA, while the development of PIS after EVAR for TAA has been less frequently observed [6]. It is of interest that our patient developed also a progressive thrombocytopenia of apparent unknown origin which is not frequently described so far in PIS. It is possible that the inflammatory response characterizing PIS could have suppressed the bone marrow activity and reduced blood cells survival as described for anemia in chronic diseases [13]. To this regard, a possible role of the preoperative inflammatory state present in our patient in the severe reaction observed after TEVAR could be suggested. Indeed TNF-*α* and IL-6 plasma levels in our patient were increased, possibly confirming this hypothesis. The increased plasma levels of laboratory inflammatory parameters observed in our patient are similar to those reported in previous series of patients who developed PIS after EVAR for AAA [3], confirming the observation that the activation of a severe inflammatory response might be an early signal for the development of PIS after EVAR [3, 4, 12]. It is still debatable if the inflammatory response after EVAR is an epiphenomenon or has a pathogenetic role in the development of the clinical consequences of PIS. To this regard, it seems that the excessive activation of the inflammatory response together with the release of inflammatory cytokines seems to have a direct role in the extensive endothelial damage, possibly leading to severe complications as well as pulmonary dysfunction, cardiovascular events, and renal and multiorgan failure [3]. Less clear is the clinical meaning, if any, of the thrombocytopenia observed in our patient who developed PIS after TEVAR. Interestingly, in the series reported by Arnaoutoglou et al., one-third of patients who underwent EVAR for AAA and experienced recurrent PIS after hospital discharge developed thrombocytopenia postoperatively [3]. Although at the moment the reason for thrombocytopenia is not known, it is possible to hypothesize that the complex inflammatory response characterizing PIS might have determined an interference with the action of thrombopoietin within the bone marrow.

The pharmacological treatment of thrombocytopenia in the present case was empirically based on corticosteroids but we observed poor effects on platelet count. Interestingly, in a recent paper by de la Motte et al., it has been reported that preoperative treatment of patients undergoing EVAR with methylprednisolone attenuates the inflammatory response with a faster recovery after EVAR for AAA [14]. Our patient underwent TEVAR in emergency, so that there was no time to schedule a preoperative corticosteroid treatment. To this regard, danazol has been successfully used in the treatment of immune and idiopathic thrombocytopenic purpura (ITP) and in thrombocytopenia related to myelodysplastic syndrome (MDS) [10, 11]. Responses are often durable and toxicity and costs are modest [10]. Indeed platelet count in our patient progressively increased after the therapy with danazol: after 12 days of therapy 1 with danazol 200 mg/tid, platelet count has almost doubled, since there was an increase of 96% in the number of platelets (26,000/*μ*L→53,000/*μ*L). The mechanism of action of danazol in MDS and in ITP remains unclear. It has been hypothesized that danazol, similar to the stimulatory effects of androgens on erythropoietin, could increase thrombopoietin plasma levels or reduce the level of antiplatelet antibodies as observed in ITP [10, 15].

In conclusion, patients undergoing TEVAR should be monitored for the development of PIS and we recommend careful monitoring of postoperative blood inflammatory markers and blood cell count above all leukocytes and platelets. The role of the different therapeutical approaches of PIS is still empiric, with reported beneficial effects with the use of NSAIDs, corticosteroids, and danazol. The possible beneficial effects of eltrombopag, a novel agonist of the thrombopoietin receptor which promotes megakaryopoiesis similar to endogenous human thrombopoietin [16], have not been evaluated yet but could represent a novel fascinating therapeutic option.
